# Quantifying the Cerebral Hemometabolic Response to Blood Transfusion in Pediatric Sickle Cell Disease With Diffuse Optical Spectroscopies

**DOI:** 10.3389/fneur.2022.869117

**Published:** 2022-07-01

**Authors:** Seung Yup Lee, Rowan O. Brothers, Katherine B. Turrentine, Ayesha Quadri, Eashani Sathialingam, Kyle R. Cowdrick, Scott Gillespie, Shasha Bai, Adam E. Goldman-Yassen, Clinton H. Joiner, R. Clark Brown, Erin M. Buckley

**Affiliations:** ^1^Wallace H. Coulter Department of Biomedical Engineering, Georgia Institute of Technology and Emory University, Atlanta, GA, United States; ^2^Department of Electrical and Computer Engineering, Kennesaw State University, Marietta, GA, United States; ^3^Pediatric Biostatistics Core, Emory University School of Medicine, Atlanta, GA, United States; ^4^Department of Pediatrics, Emory University School of Medicine, Atlanta, GA, United States; ^5^Aflac Cancer & Blood Disorders Center, Children's Healthcare of Atlanta, Atlanta, GA, United States; ^6^Children's Research Scholar, Children's Healthcare of Atlanta, Atlanta, GA, United States

**Keywords:** diffuse correlation spectroscopy (DCS), near-infrared spectroscopy, sickle cell disease (SCD), blood transfusion, cerebral hemometabolics, cerebral oxygen extraction fraction, frequency-domain near-infrared spectroscopy, cerebral blood flow

## Abstract

Red blood cell transfusions are common in patients with sickle cell disease who are at increased risk of stroke. Unfortunately, transfusion thresholds needed to sufficiently dilute sickle red blood cells and adequately restore oxygen delivery to the brain are not well defined. Previous work has shown that transfusion is associated with a reduction in oxygen extraction fraction and cerebral blood flow, both of which are abnormally increased in sickle patients. These reductions are thought to alleviate hemometabolic stress by improving the brain's ability to respond to increased metabolic demand, thereby reducing susceptibility to ischemic injury. Monitoring the cerebral hemometabolic response to transfusion may enable individualized management of transfusion thresholds. Diffuse optical spectroscopies may present a low-cost, non-invasive means to monitor this response. In this study, children with SCD undergoing chronic transfusion therapy were recruited. Diffuse optical spectroscopies (namely, diffuse correlation spectroscopy combined with frequency domain near-infrared spectroscopy) were used to quantify oxygen extraction fraction (OEF), cerebral blood volume (CBV), an index of cerebral blood flow (CBF_i_), and an index of cerebral oxygen metabolism (CMRO_2i_) in the frontal cortex immediately before and after transfusion. A subset of patients receiving regular monthly transfusions were measured during a subsequent transfusion. Data was captured from 35 transfusions in 23 patients. Transfusion increased median blood hemoglobin levels (Hb) from 9.1 to 11.7 g/dL (*p* < 0.001) and decreased median sickle hemoglobin (HbS) from 30.9 to 21.7% (*p* < 0.001). Transfusion decreased OEF by median 5.9% (*p* < 0.001), CBFi by median 21.2% (*p* = 0.020), and CBV by median 18.2% (*p* < 0.001). CMRO_2i_ did not statistically change from pre-transfusion levels (*p* > 0.05). Multivariable analysis revealed varying degrees of associations between outcomes (i.e., OEF, CBF_i_, CBV, and CMRO_2i_), Hb, and demographics. OEF, CBF_i_, and CBV were all negatively associated with Hb, while CMRO_2i_ was only associated with age. These results demonstrate that diffuse optical spectroscopies are sensitive to the expected decreases of oxygen extraction, blood flow, and blood volume after transfusion. Diffuse optical spectroscopies may be a promising bedside tool for real-time monitoring and goal-directed therapy to reduce stroke risk for sickle cell disease.

## Introduction

Sickle cell disease (SCD) is an autosomal recessive blood disorder affecting millions of people worldwide, with ~300,000 newborns diagnosed each year (1). In SCD, a single gene mutation causes production of abnormal hemoglobin, hemoglobin S (HbS) (1). In addition to impaired oxygen-carrying ability, HbS rapidly forms intracellular polymers in the deoxygenated state, distorting red blood cells (RBC) into a characteristic sickle shape (1). Sickling, decreased RBC deformability, and increased cell aggregation and hemolysis, result in increased blood viscosity and decreased microvascular perfusion (1–3). The culmination of impaired oxygen-carrying capacity and altered blood flow dramatically increases the incidence of vascular occlusion and stroke in patients with sickle cell disease (1).

Currently, routine screening with transcranial Doppler ultrasound (TCD) is employed to minimize stroke risk in children with sickle cell disease (4). Patients with persistently elevated TCD blood flow velocities of the large feeding arteries to the brain are placed on chronic transfusion therapy (CTT) to reverse metabolic stress caused by anemia and to prevent vaso-occlusion and stroke (5). CTT involves periodic RBC transfusion to dilute sickle red blood cells (sRBC) with the general goal of maintaining HbS <30% and hemoglobin (Hb) >10 g/dL (5). CTT has drastically reduced the incidence and severity of both overt stroke and silent infarction (92 and 58% relative risk reduction, respectively) (6, 7). However, recurrent infarctions occur in 23–45% of patients receiving CTT, despite successful maintenance of clinical thresholds for HbS and Hb (8–10), suggesting that current standardized CTT guidelines are insufficient for infarct prevention in a substantial fraction of patients.

Transfusion is associated with a reduction in oxygen extraction fraction (OEF) and cerebral blood flow (CBF), both of which are abnormally increased in SCD patients due to reduced arterial oxygen content (C_a_O_2_) (11, 12). These reductions are thought to alleviate hemometabolic stress by improving the brain's ability to respond to increased metabolic demand, thereby reducing susceptibility to ischemic injury (11, 12). Monitoring the cerebral hemometabolic response to transfusion may enable individualized management of transfusion thresholds, with the goal of sufficiently restoring cerebrovascular reserve by diluting or replacing sickle red blood cells. In patients for whom CTT does not adequately address metabolic needs, such monitoring may be used to identify those who would benefit from more aggressive therapies (e.g., bone marrow transplant) (12, 13).

Magnetic resonance imaging (MRI) provides excellent, high-resolution images of cerebral blood flow and oxygen extraction fraction that have been used in recent years to elucidate mechanisms underlying regional vulnerabilities to injury in patients with SCD (11, 12, 14). Indeed, recent work has demonstrated the influence of CTT on relieving hemometabolic stress in SCD patients (11, 12). However, MRI is prohibitively expensive for routine monitoring, requires sedation in children <6 y, and quantitative differences exist across scanners and software platforms. Thus, its utility in individualizing CTT management in SCD patients is limited.

Diffuse optical spectroscopies (DOS) present a low-cost, non-invasive alternative to current neuroimaging techniques like MRI. By operating in the near-infrared, DOS techniques exploit the spectral window of low absorption that exists in tissue. Photons emitted from a source placed on the tissue surface propagate several centimeters (through the scalp and skull) before reemission at the tissue surface. The properties of the detected light can be related to hemodynamic properties of the interrogated tissue, including hemoglobin oxygen saturation, blood flow, blood volume, and oxygen metabolism. While limited in depth sensitivity to the superficial cortex, DOS offers numerous advantages to other neuroimaging techniques, including portability, ease of measurements, and cost. Indeed, a handful of studies have employed a type of DOS known as continuous-wave near-infrared spectroscopy (CW-NIRS) to demonstrate the technology is sensitive to expected increases in cerebral oxygen saturation caused by transfusion (15–18). However, to date, these studies have been limited to commercially available CW-NIRS systems. While useful as a trend monitor, CW-NIRS have been shown to produce substantial inaccuracies in estimations of absolute oxygen saturation (19, 20). Thus, although these studies have observed expected trends in hemoglobin oxygen saturation following transfusion, the magnitude of these increases is likely significantly underestimated (19, 20).

Herein we employ two DOS techniques known as frequency-domain NIRS (FDNIRS) and diffuse correlation spectroscopy (DCS) to monitor the effects of blood transfusion on the brain in a cohort of children with sickle cell disease receiving CTT. FDNIRS is an alternative to CW-NIRS that has been shown to more accurately estimate hemoglobin oxygen saturation, while also providing a measurement of cerebral blood volume (CBV). DCS is a type of DOS used to measure cerebral blood flow (CBF). The combination of oxygen saturation from FDNIRS and CBF from DCS also enables us to estimate the cerebral metabolic rate of oxygen (CMRO_2_) using Fick's law (21). We hypothesize that FDNIRS combined with DCS (FDNIRS/DCS) can non-invasively quantify changes in markers of cerebral hemometabolic stress at the bedside following transfusion. Specifically, we hypothesize that FDNIRS/DCS can detect expected decreases in CBF, CBV, and OEF post-transfusion, while CMRO_2_ will remain unchanged. Further, we hypothesize that CBF, CBV, and OEF will be inversely associated with hemoglobin.

## Methods

### Patient Population

Children ages 2 to 18 years old with SCD (HbSS or HbS thalassemia) receiving chronic transfusion therapy were recruited for this study. RBC transfusions were administered by simple infusion, partial manual exchange, or apheresis following our institution's standard practice guidelines for CTT. Volume of transfusion was calculated to target a post-transfusion venous hemoglobin (Hb) of ~11 g/dL based on Hb levels measured between 1–72 h (mean 5 h) prior to transfusion. Patients were excluded on the basis of: significant illness within one month of participation, hypertension, neurologic disorder not related to SCD (e.g., seizures), Moyamoya or previous revascularization surgery, prior history of major head injury requiring a visit to an emergency department, and/or prior curative therapy. All patients and/or their legal guardians provided informed assent/consent. The study was approved by the Institutional Review Board of Emory University.

### Clinical and Laboratory Data

Complete blood count [hematocrit and hemoglobin (Hb)] and hemoglobin electrophoresis (Hb S%, HbA%) were performed on a venous blood sample obtained <72 h before transfusion. Measurements were repeated immediately after transfusion. Transcutaneous oxygen saturation (SpO_2_, %) and heart rate (HR, bpm) were measured with pulse oximetry on the index finger immediately before and after transfusion. Non-invasive cuff blood pressure was also measured before and after transfusion. C_a_O_2_ (mL/dL) was estimated from SpO_2_ and Hb, *C*_*a*_*O*_2_ = 1.39 × *SpO*_2_ × *Hb*.

### FDNIRS/DCS Experimental Protocol and Analysis

To assess the cerebral hemometabolic effects of transfusion, brief (~15 min) FDNIRS/DCS measurements were obtained within 2 h prior to the start of transfusion and were repeated at the cessation of transfusion. Due to limitations in software acquisition capabilities with our current system, FDNIRS and DCS data sets were taken sequentially, not simultaneously. For each measurement, we manually held an optical sensor over the right and left frontal cortex for 3–5 s intervals. The sensor was repositioned three times per hemisphere per optical modality (FDNIRS, DCS). After confirming that there were no significant hemispheric differences, all repetitions were averaged to yield a global mean of each measured parameter.

In the following sections, we detail the instrumentation utilized as well as the data analysis pipeline for both FDNIRS and DCS.

#### Frequency-Domain Near-Infrared Spectroscopy (FDNIRS)

FDNIRS enables quantification of oxy- and deoxy-hemoglobin concentrations (HbO and HbR, respectively, μM) of the interrogated tissue. In this study, we employed a customized FDNIRS system (Imagent, ISS Inc.) with eight near-infrared laser diode sources (690, 730, 750, 775, 785, 800, 825, and 830 nm) modulated at 110 MHz and rapidly multiplexed at 20.8 Hz, along with four photomultiplier tube detectors with gain modulation of 110 MHz + 5 kHz for heterodyne detection at 5 kHz. The patient interface consisted of a 3D-printed rigid black sensor containing five 2.5 mm fiber bundles (50 μm multimode fibers, NA = 0.66, FTTIIG23767, FiberOptics Technology, Pomfret, Connecticut), one of which was used as the source, and the other four of which were used as detectors spaced 2.0, 2.5, 3.0, and 3.5 cm from the source.

For FDNIRS data acquisition, AC amplitude and phase data for each wavelength, λ, were acquired for 3-5s at 20Hz. Measured AC amplitude attenuation, AC(r, λ), and phase shift, θ(r, λ), at each separation, r, and wavelength, λ, were first averaged over the 3–5 s acquisition interval and discarded if the phase standard deviation exceeded 5° or if the AC coefficient of variation was > 10%. Averaged data were then fit to the semi-infinite solution to the diffusion equation to extract wavelength-dependent reduced scattering and absorption coefficients ($\mu_{s}^{\prime }\left(\lambda \right)$ and μ_a_(λ), respectively) (22, 23). This approximation assumes a linear relationship between ln(*AC*(*r*, λ) × *r*^2^) and θ(*r*, λ) vs. r. To ensure the data fit this linear model, we calculated Pearson's correlation coefficient, R, for both of these relationships, and we discarded data for a given λ in which R^2^ <0.97. The entire dataset was discarded if <5 wavelengths passed this linear fit criterion or if the slope of $\mu_{s}^{\prime }\left(\lambda \right)$ vs. λ was > 0.

Estimates of HbO and HbR were obtained by fitting μ_a_(λ) vs. λ to the hemoglobin spectrum (24). Next, HbO and HbR were used to derive total hemoglobin concentration (HbT = HbO + HbR), hemoglobin oxygen saturation (SO_2_ = HbO/HbT × 100%), oxygen extraction fraction (OEF = (SpO_2_ – SO_2_)/ (γ × SpO_2_), and cerebral blood volume [CBV = MW × HbT/(D_bt_ × Hb)]. Here, γ is the fraction of blood volume within the probed venous compartment of the tissue (assumed to be 1 for all subjects for simplicity), MW = 64,500 g/mol is the molecular weight of hemoglobin, and D_bt_ is the brain tissue density (assumed to be 1.05 g/mL).

Additionally, because DCS data were obtained at 852 nm to quantify blood flow, we extrapolated FDNIRS-measured $\mu_{s}^{\prime }$ and μ_*a*_ to 852 nm. To estimate $\mu_{s}^{\prime }\left(852nm\right)$, a linear model was fit to $\mu_{s}^{\prime }\left(\lambda \right)$ vs. λ and extrapolated to 852 nm. To estimate μ_*a*_(852*nm*), we used the following formula:


(1) $$\begin{array}{r} \begin{array}{c} \begin{array}{l} \mu_{a}(852nm)=\varepsilon_{HbO}(852nm)\times HbO+\varepsilon_{HbR}(852nm)\times HbR\;\\ +0.75\times \mu_{a,water}(852nm) \end{array} \end{array} \end{array}$$

where HbO and HbR were obtained from FDNIRS ε_*HbO*_ and ε_*HbR*_ are the extinction coefficients of oxy- and deoxyhemoglobin, and the factor of 0.75 is the assumed water content of the tissue.

#### Diffuse Correlation Spectroscopy

Diffuse correlation spectroscopy quantifies temporal fluctuations in reflected light intensity measured by a detector located some fixed distance away from a near-infrared light source placed on the tissue surface. These intensity fluctuations, which are caused by the motion of red blood cells, can be used estimate an index of blood flow in the underlying tissue. The DCS system employed herein consisted of an 852 nm long-coherence laser (iBeam Smart, TOPTICA Photonics, Farmington, New York), two four-channel single photon counting modules (SPCMQA4C-IO, Perkin-Elmer, Quebec, Canada), an eight-channel hardware correlator (Flex05-8ch, New Jersey), and a counter/timer data acquisition board (PCIe-6612, National Instruments) (25). Detected photon counts were used to estimate an intensity autocorrelation function, *g*_2_(τ), either *via* the hardware correlator or with a custom software correlator. For the first 26 transfusion events, we used the hardware correlator; for the remaining transfusion events we transitioned to the software correlator to increase versatility of data acquisition [e.g., acquisition rate, temporal resolution of *g*_2_(τ)]. *In vitro* phantom validations were used to confirm that the hardware correlator and the software correlator yielded the same flow index, as demonstrated by Wang *et al*. (25).

The patient interface for DCS measurements consisted of a black sensor containing one 800 μm multimode source fiber (FT800EMT, Thorlabs) and seven single-mode detector fibers (780-HP, Thorlabs) bundled together and spaced 2.5 cm from the source. Compliance with the American National Standards Institute (ANSI) maximum permissible exposure of skin to laser radiation (<4 mW/mm^2^ at 852 nm) was ensured by utilizing a 5mm right-angle prism (MRA03-E03, Thorlabs) to couple light from the source fiber to the tissue surface, thereby yielding a spot size radius >2 mm, and by adjusting the laser power so that the output at the tissue surface was <50 mW.

For DCS data acquisition, hardware correlator data was acquired at 1Hz. Software correlator data was acquired at 20 Hz and downsampled by averaging to 1 Hz prior to analysis for consistency. Thus, each measurement repetition consisted of 3–5 data points. Data points with intensity <6 kHz were excluded from analysis to avoid g_2_(τ) with poor signal-to-noise ratio (SNR).

To estimate CBF_i_, the measured intensity autocorrelation functions, g_2_(τ), were first averaged across the 7 detector channels to improve SNR. Averaged curves were fit to the semi-infinite solution of the correlation diffusion equation to derive CBF_i_ (24). When FDNIRS data was available, measured μ_a_ and $\mu_{s}^{{}^{\prime }}$ at 852 nm were incorporated into the fit for CBF_i_. When unavailable (as was the case in 2 measurements), μ_*a*_ and $\mu_{s}^{{}^{\prime }}$ were assumed to be 0.16 cm^−1^ and 8.4 cm^−1^, respectively (26). Fits were discarded if ϵ>14% where $\epsilon =\underset{i}{\sum }\left(g_{2,\;fit}\left(\tau_{i}\right)-g_{2,\;meas}\left(\tau_{i}\right)\right)/g_{2,\;fit}\left(\tau_{i}\right)\times 100\%.$ As a final quality control step, repetitions with < 3 data points were discarded, and entire measurement sessions were discarded if < 2/6 repetitions failed to meet quality control criteria.

To estimate CMRO_2i_, we employed a derivative of Fick's law that relates CMRO_2i_ to measurable quantities by assuming a compartmentalized model of the vasculature (24, 27):


(2) $$\begin{array}{r} CMRO_{2i}=OEF\;\times \;CBF_{i}\;\times \;C_{a}O_{2} \end{array}$$

### Statistical Analysis

Primary analyses were performed at the transfusion level, with a total of 35 transfusions. Summary statistics are expressed as median (interquartile range: 1^st^ quartile, 3^rd^ quartile) for continuous measurements and count (percentage) for categorical measurements. Due to the relatively small sample size, non-parametric Wilcoxon signed-rank test for paired data was used to compare pre- and post-transfusion measures. Linear mixed-effects regression models with random-intercept at the transfusion level were used to examine the association of each outcome measure (OEF, CBF_i_, CBV, and CMRO_2i_) with demographical (sex and age) and laboratory data (Hb, HbA and HbS). These models were two-level and accounted for repeated measures (pre- and post-transfusion measurements) nested within the same transfusion event. Selection of predictors in the final multivariable model was performed by backward selection using marginally significant predictors from bivariable results with a threshold of p-value < 0.1. We used variance inflation factors to assess collinearity among predictors included in multivariable models and found a certain degree of collinearity among Hb, HbA, and HbS. In these scenarios, Hb was chosen as the representative predictor, since Hb is generally accepted as the clinical parameter to manage RBC transfusion volume. When the slope estimate of a certain predictor was a small decimal number with too many trailing zeros, the predictor was linearly scaled by a simple conversion (e.g., 0.1 × HbS) to produce an easily readable and interpretable slope estimate. As a secondary analysis, a three-level linear mixed-effect model was fit for the 10 patients who had longitudinal measurements from repeated transfusions, where pre- and post-transfusion were nested within each transfusion event, and multiple transfusions were nested within the patient. Due to the limited sample size, results from this analysis are for preliminary information only and should be6g interpreted with care. Further, ordinary least square regression was used to examine the association of the relative change in each outcome measure (rOEF, rCBF_i_, rCBV, and rCMRO_2i_) with demographical data (sex and age) and changes in laboratory data from pre- to post-transfusion (dHb, dHbA and dHbS). Relative changes in outcome measures were defined as the percent change in outcome measure from pre- to post-transfusion (e.g., rOEF = (OEF_post_ – OEF_pre_)/OEF_pre_ × 100%); changes in laboratory data were defined as the difference in laboratory data from pre- to post- transfusion (e.g., dHb = Hb_post_ – Hb_pre_). All statistical analyses were performed with CRAN R (version 3.4.1, R Foundation). Significance was assessed at the 0.05 level.

## Results

### Patient Characteristics

A total of 23 children with HbSS on chronic transfusion therapy were enrolled. Patients were mostly female (17/23, 73.9%). Of these 23 patients, 8 (34.8%) were monitored on 2 separate transfusions, and 2 (8.7%) were monitored on 3 separate transfusions. Each follow-up measurement was performed > 6 months after the prior transfusion. In total, we measured 35 transfusion events. Of these 35 transfusions, 30 (85.7%) were simple, 4 (11.4%) were partial exchange, and one (2.9%) was apheresis (Table 1). At the time of transfusion, patients had a median (IQR) age of 13.9 (10.7, 15.9) years, and 23% were reported to be receiving hydroxyurea therapy.

**Table 1:** Patient characteristics (*n* = 35).

Age (y)	13.9 (10.7, 15.9)
Height (cm)	158.0 (147.8, 161.9)
Weight (kg)	55.7 (42.4, 69.6)
**Type of transfusion, no. (%)**	
Simple	30 (85.7 %)
Partial exchange	4 (11.4 %)
Apheresis	1 (2.8 %)
Hydroxyurea, no. (%)	8 (22.9 %)
Duration on CTT, (mo)	16.1 (8.3, 21.3)

*Data are reported as median (interquartile range) unless stated otherwise*.

### Effect of Transfusion on Hb and Patient Vitals

Tranfusion significantly increased Hb by median 2.9 (IQR: 1.8, 3.3) g/dL (*p* < 0.001), HbA by median 10.5 (7.9, 17.2) % (*p* < 0.001), and decreased HbS by median 8.0 (14.3, 6.8) % (*p* < 0.001, Table 2). Heart rate decreased significantly by median 8.0 (15.0, 2.3) bpm (*p* < 0.001) after transfusion, consistent with the known adaptive response from improved oxygen delivery and cardiac output. SpO_2_ and MAP remained statistically unchanged.

**Table 2:** Effects of transfusion on hemoglobin and patient vitals.

	**Pre-transfusion**	**Post-transfusion**	**Difference in Post- and Pre-transfusion**	***p***
Hb (g/dL)	9.1 (8.3, 10.0), 35	11.7 (11.3, 12.3), 31	2.9 (1.8, 3.3), 31	**<** **0.001**
HbA (%)	61.9 (51.1, 71.2), 34	74.2 (65.8, 81.8), 28	10.5 (7.9, 17.2), 27	**<** **0.001**
HbS (%)	30.9 (22.5, 39.7), 34	21.7 (15.3, 27.2), 28	−8.0 (−14.3, −6.8), 27	**<** **0.001**
C_a_O_2_ (mL/dL)	12.4 (11.6, 13.8), 35	16.3 (15.5, 17.1), 31	4.0 (2.5, 4.6), 31	**<** **0.001**
SpO_2_ (%)	100 (99, 100), 35	100 (99, 100), 35	0.0 (0.0, 0.8), 35	0.75
HR (bpm)	91.0 (79.3, 98.5), 35	82.0 (73.3, 90.9), 35	−8.0 (−15.0, −2.3), 35	**<** **0.001**
MAP (mmHg)	80.3 (76.4, 86.9), 35	80.0 (74.3, 85.4), 35	−3.0 (−7.7, 5.3), 35	0.29

*Data are reported as median (interquartile range), count of non-missing values. p-values were obtained from a two-sided Wilcoxon signed-rank test. Hb, hemoglobin; HbA, hemoglobin A; HbS, hemoglobin S; CaO_2_, arterial oxygen concentration; SpO_2_, transcutaneous oxygen saturation; HR, heart rate; MAP, mean arterial blood pressure. The bold values indicate the value of p < 0.05 which are statistically significant*.

### Effect of Transfusion on Cerebral Hemometabolics

**Figure 1:**
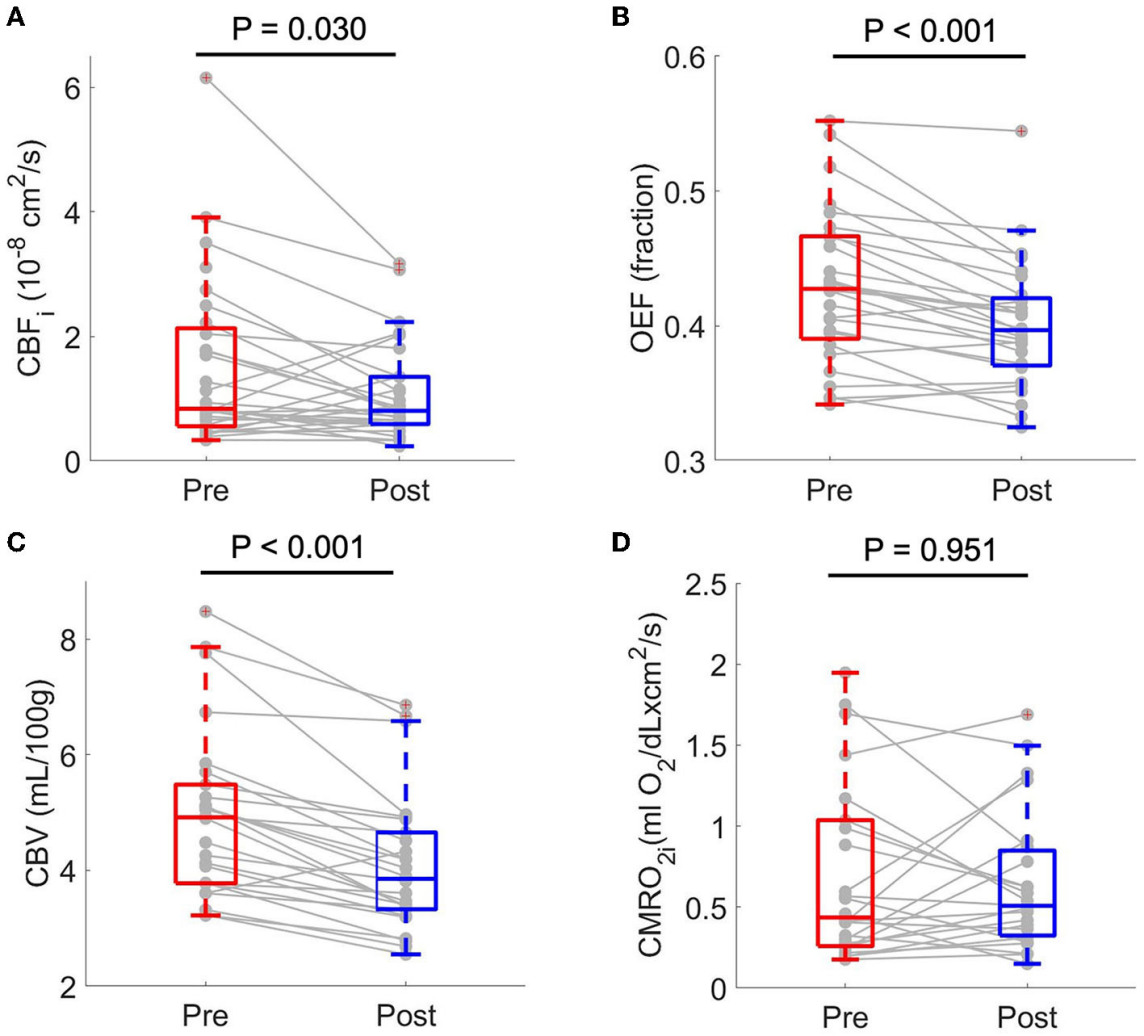
Boxplots of paired pre- and post-transfusion cerebral blood flow index, CBF_i_ **(A)** oxygen extraction fraction, OEF **(B)** cerebral blood volume, CBV **(C)** and cerebral metabolic rate of oxygen index, CMRO_2i_ **(D)**. For each boxplot, the central line denotes the median and the bottom and top edges of the box indicate the 25^th^ and 75th percentiles, respectively. The whiskers extend to the most extreme data points not considered outliers. Individual connected dots represent each pre- and post-transfusion matched pair. *p*-values were obtained from two-sided paired Wilcoxon signed rank tests. CMRO_2i_ scaled by a factor of 10^7^.

**Figure 2:**
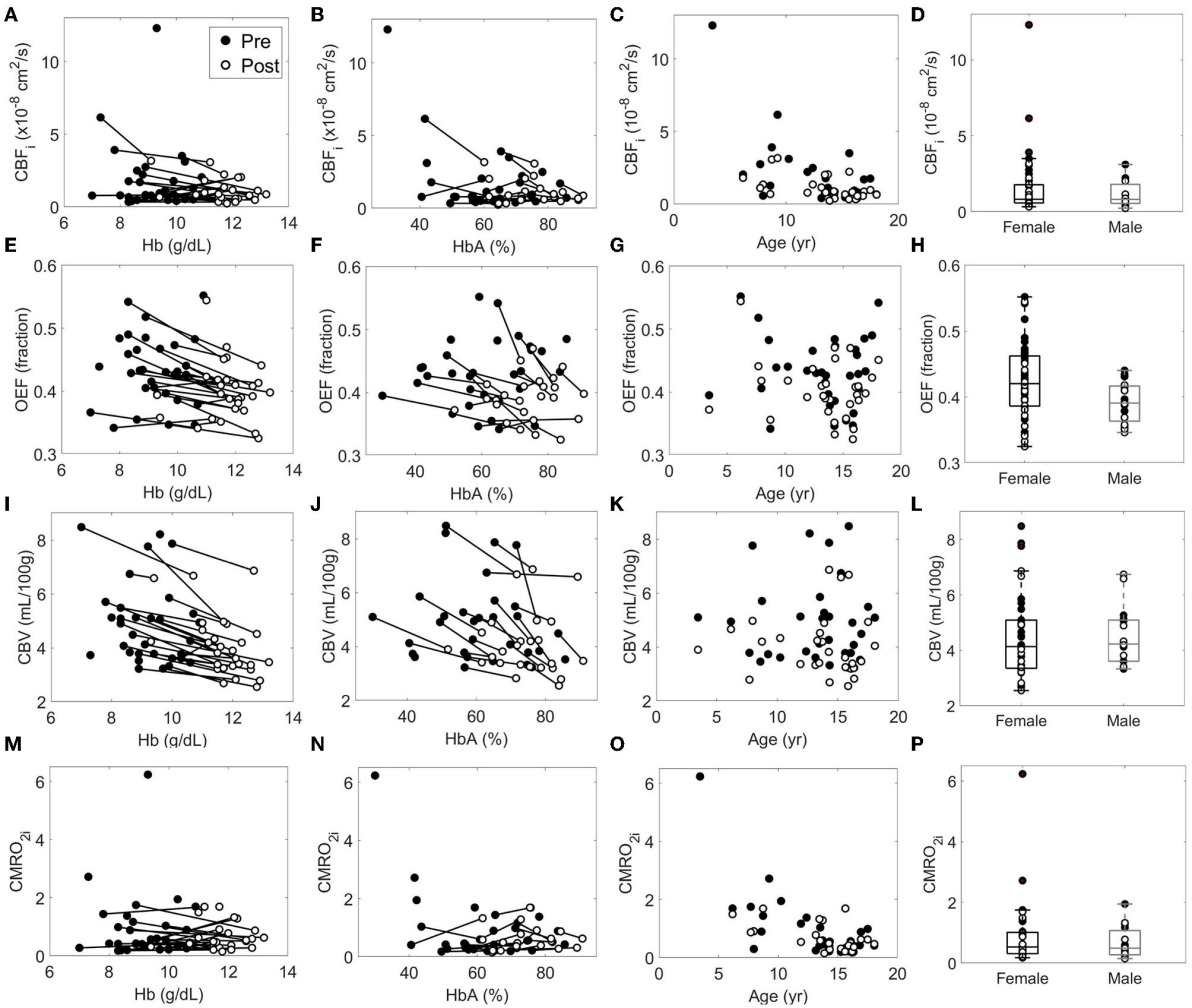
Relationship between each cerebral hemometabolic parameter (**A–D**: CBF_i_, **E–H**: OEF, **I–L**: CBV and **M–P**: CMRO_2i_) and hemoglobin (Hb), hemoglobin A (HbA), age, and sex. In each subplot, solid circles represent pre-transfusion data and hollow circles represent post-transfusion data. Solid lines connect pairs of pre- and post-transfusion data from a single measurement session; solid lines were not included for age and gender panels to aide in visualization, as neither age nor sex changed during pre-post transfusion. CMRO_2i_, cerebral metabolic rate of oxygen, in units of 10^−7^ mL O_2_/dL × cm^2^/s.

Out of 70 FDNIRS/DCS measurements, 62 FDNIRS (89%) and 62 DCS (89%) measurements passed quality control criteria to be included in analysis. Transfusion was associated with significant decreases in CBF_i_, OEF, and CBV. CBF_i_ decreased by median 21.5 (46.1, −16.1) % (*p* = 0.030), OEF decreased by median 5.9 (10.1, 2.9) % (*p* < 0.001), and CBV decreased by median 18.2 (22.8, 10.2) % (*p* < 0.001). CMRO_2i_ did not statistically change in response to transfusion (*p* > 0.05, Figure 1, Table 3). An outlier in both CBF_i_ and CMRO_2i_ made pre-/post- transfusion trends difficult to visualize in the subplots of Figure 2. Supplementary Figure 1 shows this same data with outliers removed. Note, these outliers were included in all analyses; secondary analysis performed by removing outliers did not change results.

**Table 3:** Effects of transfusion on blood flow, oxygen extraction, blood volume, and oxygen metabolism.

	**Pre-transfusion**	**Post-transfusion**	**Relative change (%)**	***p***
CBF_i_ (10^−8^ cm^2^/s)	0.84 (0.56, 2.13), 32	0.82 (0.60, 1.35), 30	−21.5 (−46.5, 16.1), 29	**0.030**
OEF (fraction)	0.43 (0.40, 0.47), 32	0.40 (0.37, 0.42), 30	−5.9 (−10.1, −2.9), 28	**<0.001**
CBV (mL/100g)	4.7 (3.8, 5.4), 32	3.9 (3.3, 4.6), 28	−18.2 (−22.8, −10.2), 26	**<0.001**
CMRO_2i_	0.46 (0.28, 1.15), 31	0.53 (0.34, 0.90), 24	13.8 (−36.3, 82.3), 23	0.951

*Data are reported as median (interquartile range), count of non-missing values. p-values were obtained from a two-sided Wilcoxon signed-rank test. CBFi, cerebral blood flow index; OEF, oxygen extraction fraction; CBV, cerebral blood volume; CMRO_2i_, cerebral metabolic rate of oxygen, in units of 10^−7^ mL O_2_/dL × cm^2^/s. The bold values indicate the value of p < 0.05 which are statistically significant*.

Bivariable relationships between predictors (Hb, HbA, HbS, age, and sex) and cerebral hemometabolic outcomes (CBF_i_, OEF, CBV, and CMRO_2i_) are visualized in Figure 2, and linear regression models for bivariable and multivariable relationships are presented in Table 4. Bivariably, CBF_i_ was inversely associated with age (*p* < 0.001), HbA (*p* = 0.011), and HbS (*p* = 0.016). A marginally significant inverse association between CBF_i_ and Hb was also observed (*p* = 0.053). In multivariable models, age and Hb, as well as age and HbA, remained significant independent predictors of CBF_i_. For OEF, significant inverse associations were observed with Hb (*p* < 0.001) and HbA (*p* = 0.008); additionally, OEF was significantly positively associated with HbS (*p* = 0.024). OEF was marginally significantly higher in females than in males (*p* = 0.059). In multivariable analysis, Hb, as well as sex and HbA, remained significant independent predictors of OEF (Hb, HbS and HbA were not modeled together due to multicollinearity). For CBV, bivariable analysis revealed significant inverse associations with Hb (*p* < 0.001) and HbA (*p* < 0.001) and a positive association with HbS (*p* = 0.002). For CMRO_2i_, age was the only significant correlate (*p* < 0.001). While the current analysis employed 2-level hierarchical models at the transfusion-level, nesting pre-post observations within transfusions, we also performed a sensitivity analysis wherein repeated transfusions were also nested within patients, resulting in 3-level hierarchical models. The only meaningful change in the results from Table 4 observed in this 3-level analysis was a significant inverse association between HbA and CMRO_2i_ (*p* = 0.023, Supplementary Table 1).

**Table 4:** Bivariable and multivariable analysis of factors influencing cerebral hemometabolic parameters.

	**Bivariable**		**Multivariable**			
	**Est (95% CI)**	***p***	**Est (95% CI)^a^**	***p***	**Est (95% CI)^b^**	***p***
***CBF**_***i***_*						
Age (y)	−0.35 (−0.49, −0.21)	**<0.001**	−0.35 (−0.49, −0.22)	**<0.001**	−0.31 (−0.45, −0.18)	**<0.001**
Sex	0.17 (−1.49, 1.82)	0.847	-		-	
Hb	−0.13 (−0.26, 0.00)	0.053	−0.14 (−0.27, −0.01)	**0.040**	-	
HbA% (× 0.1)	−0.36 (−0.67, −0.09)	**0.011**	-		−0.29 (−0.56, −0.04)	**0.028**
HbS% (× 0.1)	0.38 (0.08, 0.72)	**0.016**	-		-	
***OEF***						
Age (y, ×0.01)	−0.17 (−0.65, 0.31)	0.493	-		-	
Sex	0.04 (0.00, 0.07)	0.059	0.03 (−0.01, 0.07)	0.107	0.04 (0.00, 0.08)	**0.045**
Hb (× 0.1)	−0.11 (−0.14, −0.08)	**<0.001**	−0.11 (−0.14, −0.07)	**<0.001**	-	
HbA% (× 0.01)	−0.11 (−0.20, −0.02)	**0.008**	-		−0.12 (−0.20, −0.03)	**0.005**
HbS% (× 0.01)	0.11 (0.00, 0.21)	**0.024**	-		-	
***CBV***		
Age (y, ×0.1)	−0.15 (−1.45, 1.14)	0.818	-		-	
Sex	0.03 (−1.03, 1.08)	0.961	-		-	
Hb	−0.33 (−0.41, −0.25)	**<0.001**	-		-	
HbA% (× 0.1)	−0.49 (−0.70, −0.27)	**<0.001**	-		-	
HbS% (× 0.1)	0.51 (0.23, 0.76)	**0.002**	-		-	
***CMRO**_**2*i***_*		
Age (y)	−0.21 (−0.29, −0.14)	**<0.001**	-		-	
Sex	0.27 (−0.64, 1.17)	0.570	-		-	
Hb (× 0.1)	−0.10 (−0.74, 0.51)	0.745	-		-	
HbA% (× 0.01)	−0.21 (−2.00, 1.11)	0.741	-		-	
HbS% (× 0.01)	0.06 (−1.37, 1.88)	0.933	-		-	

*CBFi, cerebral blood flow index; Hb, hemoglobin; HbA%, percent hemoglobin A out of total hemoglobin; HbS%, percent hemoglobin S out of total hemoglobin; OEF, oxygen extraction fraction; CBV, cerebral blood volume; CMRO_2i_, cerebral metabolic rate of oxygen. Hb variables (Hb, HbA %, HbS % were not modeled together due to multicollinearity*;

a *Multivariable models considering Hb with other predictors*;

b *Multivariable models considering HbA with other predictors. The bold values indicate the value of p < 0.05 which are statistically significant*.

**Figure 3:**
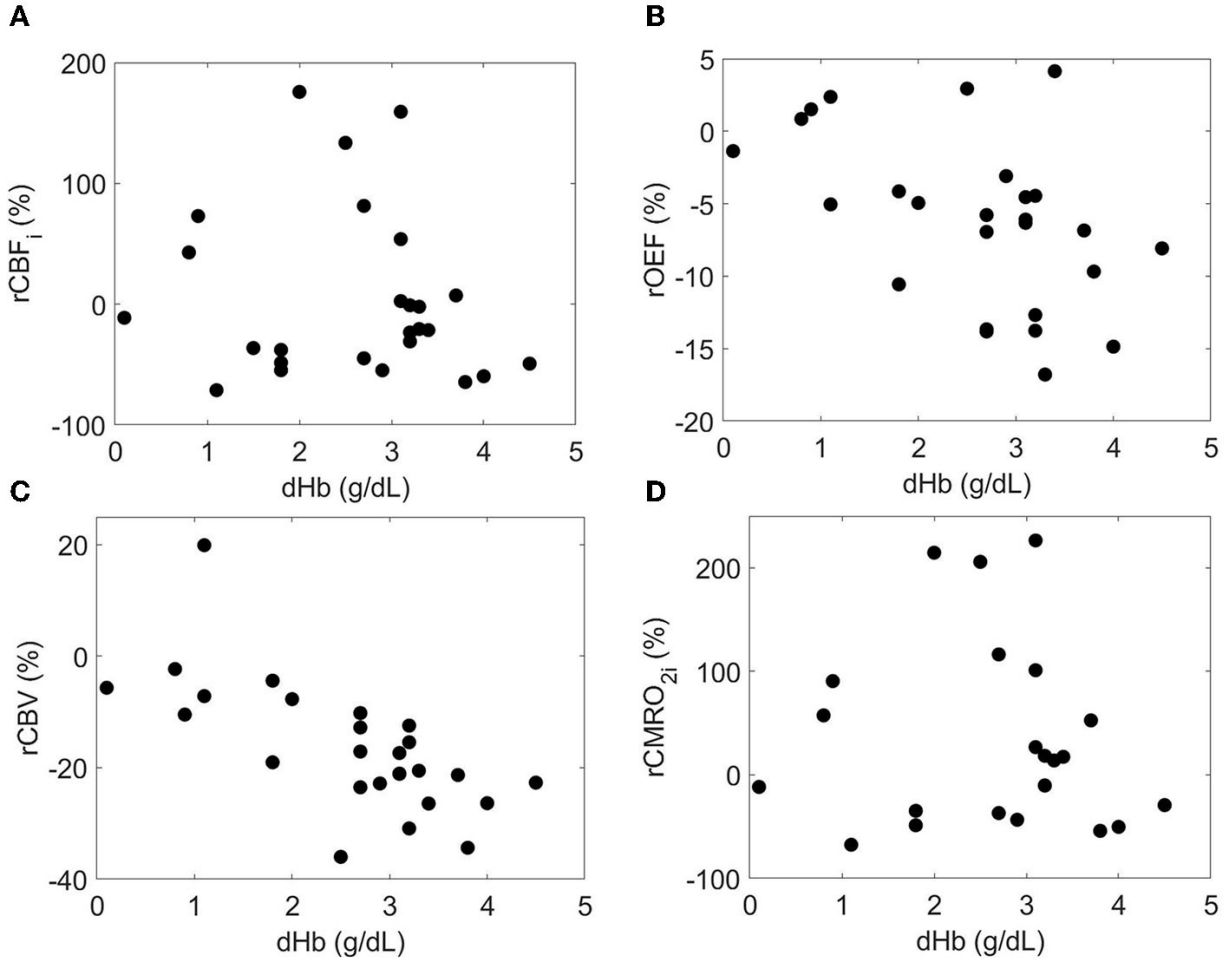
Relationship between the relative each cerebral hemometabolic parameter due to transfusion (**A**: rCBF_i_, **B**: rOEF, **C**: rCBV and **D**: rCMRO_2i_) and the change in hemoglobin (dHb).

Finally, bivariable models were used to investigate the relationship between predictor variables and relative changes in each cerebral hemometabolic outcome from pre- to post-transfusion (Table 5, Figures 3A-D). Relative change in OEF was inversely associated with dHb (*p* = 0.010, Figure 3B). Similarly, relative change in CBV was also inversely associated with dHb (*p* < 0.001, Figure 3C). Relative change in CBF_i_ and CBV were significantly higher in males versus females (*p* = 0.007 and 0.040, respectively).

**Table 5:** Bivariable analysis of factors influencing changes in cerebral hemometabolic parameters.

	**Bivariable**	
	**Est (95% CI)**	***p***
***rCBF**_***i***_*		
Age (y)	−3.13 (−8.97, 2.71)	0.598
Sex	−75.2 (−101.09, −49.31)	**0.007**
dHb (g/dL)	−8.34 (−21.23, 4.55)	0.523
dHbA%	2.74 (0.15, 5.33)	0.303
dHbS%	−2.29 (−5.01, 0.42)	0.408
***rOEF***		
Age (y)	−0.54 (−0.83, −0.25)	0.074
Sex	−3.64 (−5.95, −1.33)	0.127
dHb (g/dL)	−2.65 (−3.60, −1.70)	**0.010**
dHbA%	0.12 (−0.11, 0.25)	0.589
dHbS%	−0.13 (−0.37, 0.11)	0.571
***rCBV***		
Age (y)	0.27 (−0.36, 0.90)	0.671
Sex	−10.03 (−14.64, −5.39)	**0.040**
dHb (g/dL)	−0.73 (−0.96, −0.57)	**<0.001**
dHbA%	0.23 (−0.12, 0.58)	0.510
dHbS%	−0.38 (−0.74, −0.02)	0.305
***rCMRO**_**2*i***_*		
Age (y)	−3.13 (−8.97, 2.71)	0.598
Sex	−73.36 (−111.26, −35.46)	0.067
dHb (g/dL)	−6.66 (−23.91, 10.59)	0.703
dHbA%	3.65 (0.04, 7.26)	0.325
dHbS%	−3.33 (−7.07, 0.40)	0.384

*rCBFi, relative % change in cerebral blood flow index; dHb, delta hemoglobin; dHbA%, delta percent hemoglobin A out of total hemoglobin; dHbS%, delta percent hemoglobin S out of total hemoglobin; rOEF, relative % change in oxygen extraction fraction; rCBV, relative % change in cerebral blood volume; rCMRO_2i_, relative % change in cerebral metabolic rate of oxygen. The bold values indicate the value of p < 0.05 which are statistically significant*.

## Discussion

Herein, we demonstrate the feasibility of using diffuse optical spectroscopies, specifically FDNIRS and DCS, as a low-cost and non-invasive means to evaluate changes in oxygen extraction fraction, cerebral blood flow, blood volume, and oxygen metabolism after transfusion in children with sickle cell disease. We found that FDNIRS/DCS measurements of OEF, CBF_i_, and CBV were significantly lower post-transfusion than pre-transfusion, while no significant changes were observed in CMRO_2i_ (Figure 1). These observations are consistent with prior neuroimaging studies using MRI and positron emission tomography that demonstrate significant whole-brain decreases in OEF, CBF, and CBV following transfusion (11, 12). Further, OEF, CBF_i_, and CBV were significantly inversely associated with hemoglobin concentration, and these associations persisted after accounting for the influence of age and sex (Table 4). While our changes in OEF largely agree with previous reports (11, 12), the decreases in CBF_i_ that we measured were larger than expected (26). We hypothesize that the origin of these differences is due to the confounding influence of hematocrit (28) and work is ongoing to develop correction factors that account for this influence.

As expected, when regressed against all cerebral hemometabolic outcome variables, age was significantly inversely correlated with CBF_i_ (Figure 2C, bivariable and multivariable analyses, Table 4) and CMRO_2i_ (Figure 2O, bivariable analysis, Table 4). However, the magnitude of the decreases in CBF_i_ and CMRO_2i_ seen with age are large compared to previous reports (29–33). These exaggerated changes may be due to age-related changes that alter extracerebral signal contributions to the measured optical signal, e.g., increases in scalp/skull thickness, vessel density, and possibly increased concentration of hemoglobin production in skull bone marrow (34).

We note that we found sex was a significant predictor of OEF after adjusting for HbA (Table 4), with females having a higher OEF than males, and that sex was significantly associated with relative changes in both CBF and CBV due to transfusion with males having a higher relative change than females (Table 5). However, these correlations have not been reported in previous studies of children with sickle cell disease (11, 12, 35) and may be an artifact of the relatively small number of males in our cohort.

Finally, we note several limitations of this study. First, hemispheric data was averaged, resulting in a single, whole-brain value for each cerebral hemometabolic marker. While this approach was justified given that no significant differences were observed between hemispheres for the cohort on a whole, marked hemispheric asymmetry was observed in a small subset of patients. Future work with an expanded cohort will explore the complex relationship between vasculopathy and asymmetrical response to transfusion. Second, for subject convenience, post-transfusion measurements were performed immediately after transfusion, which likely missed post-transfusion equilibrium. While previous studies have shown that the most significant changes in cerebral tissue oxygen saturation occur within the first 30 min to 1 h of transfusion start (17, 36), measuring prior to equilibrium could potentially blunt changes in measured cerebral hemometabolic markers (17). Third, our optical measurements are limited in sensitivity to the frontal cortex. While data from MRI suggest the effects of transfusion are global and uniform within gray matter (35), we are unable to directly comment on the whole-brain hemometabolic response to transfusion. Fourth, 11% of data was discarded due to motion artifact and/or poor SNR. With improvements in real-time analysis to ensure adequate data quality at the bedside, we anticipate that this discard rate can be dramatically reduced. Fifth, both our FDNIRS and DCS analyses make the first-order assumption that the head is a homogenous medium of uniform optical properties/flow dynamics. This oversimplification neglects the influence of extracerebral layers (skull, scalp, cerebrospinal fluid), which can be appreciable (37) and, in the case of this cohort, may be variable given the range of ages (2–18y). While the consistency of our results with those of other neuroimaging modalities suggest that this influence is not insurmountable, minimizing these contributions either through advanced analytical modeling (37–39) or through hardware approaches that isolate photons that have traveled deeper in tissue (40, 41) will be an important future step toward the clinical translation of NIRS/DCS. Finally, when estimating OEF with FDNIRS, we assumed that the fraction of our signal that arises from the venous compartment is the same across our subjects (and equal to 1, although the exact magnitude of this fractional value is less important since we assumed it was the same for everyone). Inter-participant variations in this assumed value could lead to errors in the estimation of OEF.

## Conclusion

In summary, we demonstrate that diffuse optical spectroscopies (namely, FDNIRS combined with DCS) can be used to detect expected decreases in OEF, CBV, and CBF_i_ that occur in sickle cell patients in response to blood transfusion. These results suggest that FDNIRS/DCS could be useful as a low-cost, non-invasive screening tool to enable individualized management of transfusion thresholds, and to identify patients who might benefit from a more aggressive therapy such as stem cell transplant.

## Data Availability Statement

The raw data supporting the conclusions of this article will be made available by the authors, without undue reservation.

## Ethics Statement

The study was approved by the Institutional Review Board of Emory University. Written informed consent to participate in this study was provided by the participants' legal guardian/next of kin.

## Author Contributions

EB, RCB, and CJ conceived and designed the study. SYL, ROB, KT, ES, KC, AG-Y, and AQ collected data. SYL and ROB analyzed data. SG and SB helped with statistical analysis. SYL, ROB, and EB drafted the manuscript. SYL, ROB, SG, SB, AQ, CJ, RCB, and EB edited the manuscript. All authors contributed to the article and approved the submitted version.

## Funding

This project was supported by National Institute of Health R01HL152322 (EB), R21HL138062 (EB), F31HL154703 (ES), and American Heart Association 19POST34380337 (SYL). This work was supported by the National Science Foundation Graduate Research Fellowship Program under Grant No. 1937971 (ROB).

## Author Disclaimer

Any opinions, findings, and conclusions or recommendations expressed in this material are those of the author(s) and do not necessarily reflect the views of the National Science Foundation.

## Conflict of Interest

The authors declare that the research was conducted in the absence of any commercial or financial relationships that could be construed as a potential conflict of interest.

## Publisher's Note

All claims expressed in this article are solely those of the authors and do not necessarily represent those of their affiliated organizations, or those of the publisher, the editors and the reviewers. Any product that may be evaluated in this article, or claim that may be made by its manufacturer, is not guaranteed or endorsed by the publisher.
