# Supplementary material for: Quantifying the Cerebral Hemometabolic Response to Blood Transfusion in Pediatric Sickle Cell Disease With Diffuse Optical Spectroscopies
Source: Front Neurol. 2022 Jul 1;13:869117. doi: 10.3389/fneur.2022.869117 (PMC9283827; doi:10.3389/fneur.2022.869117)
Supplement: Supplementary file 1 [file Image_1.pdf]

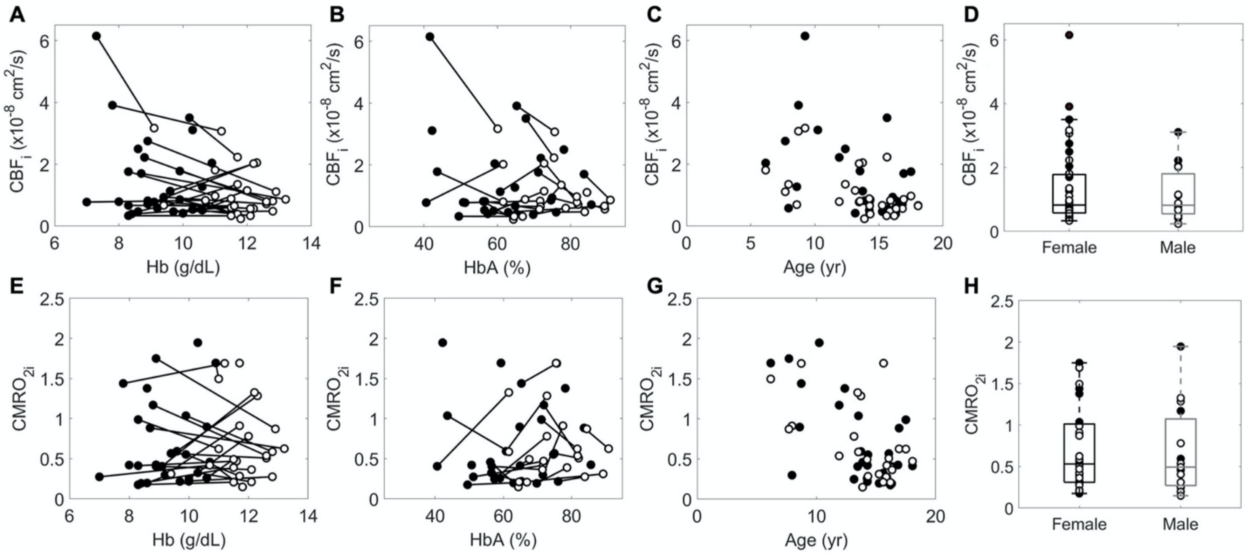

Supplemental Figure 1. Relationship between each cerebral hemometabolic parameters (A-D: CBF_i and E-H: $CMRO_{2i}$) and hemoglobin (Hb), hemoglobin A (HbA), age, and sex without outliers. In each subplot, solid circles represent pre-transfusion data and hollow circles represent post-transfusion data. Solid lines connect pairs of pre- and post-transfusion data from a single measurement session; solid lines were not included for age and gender panels to aide in visualization, as neither age nor sex changed during pre-post transfusion. $CMRO_{2i}$, cerebral metabolic rate of oxygen, in units of 10^{-7} mL O_2/dL × cm_2/s.
