# Supplementary material for: Quantifying the Cerebral Hemometabolic Response to Blood Transfusion in Pediatric Sickle Cell Disease With Diffuse Optical Spectroscopies
Source: Front Neurol. 2022 Jul 1;13:869117. doi: 10.3389/fneur.2022.869117 (PMC9283827; doi:10.3389/fneur.2022.869117)
Supplement: Supplementary file 2 [file Table_1.pdf]

Supplemental Table 1. Bivariable and multivariable analysis of factors influencing cerebral hemometabolic parameters with 3-layer hierarchical model.

	Bivariable		Multivariable			
	Est (95% CI)	*p*	Est (95% CI)[1]	*p*	Est (95% CI)[2]	*p*
CBF						
Age (y)	-0.36 (-0.52, -0.21)	**<0.001**	-0.37 (-0.52, -0.22)	**<0.001**	-0.33 (-0.48, -0.18)	**< 0.001**
Sex	-0.30 (-2.01, 2.62)	0.801	-		-	
Hb	-0.13 (-0.26, 0.01)	**0.038**	-0.14 (-0.26, -0.02)	**0.025**	-	
HbA% (×0.1)	-0.34 (-0.55, -0.15)	**0.012**	-		-0.30 (-0.49, -0.11)	**0.003**
HbS% (×0.1)	0.34 (0.11, 0.59)	**0.006**	-		-	
OEF						
Age (y, ×0.01)	-0.17 (-0.64, 0.30)	0.493	-		-	
Sex	0.04 (0.00, 0.07)	0.059	0.03 (-0.01, 0.07)	0.107	0.04 (0.00, 0.08)	0.064
Hb (×0.1)	-0.11 (-0.14, -0.08)	**< 0.001**	-0.11 (-0.14, -0.07)	**< 0.001**	-	
HbA% (×0.01)	-0.11 (-0.20, -0.02)	**0.006**	-		-0.12 (-0.20, -0.03)	**0.005**
HbS% (×0.01)	0.12 (0.00, 0.22)	**0.017**	-		-	
CBV						
Age (y, ×0.1)	0.28 (-1.12, 1.69)	0.698	-		-	
Sex	-0.17 (-1.50, 1.14)	0.796	-		-	
Hb	-0.33 (-0.41, -0.25)	**< 0.001**	-		-	
HbA% (×0.1)	-0.40 (-0.63, -0.13)	**< 0.001**	-		-	
HbS% (×0.1)	0.41 (0.12, 0.68)	**0.001**	-		-	
$CMRO_{2i}$						
Age (y)	-0.24 (-0.33, -0.15)	**< 0.001**	-		-	
Sex	0.42 (-0.82, 1.68)	0.515	-		-	
Hb (×0.1)	-0.14 (-0.77, 0.48)	0.676	-		-	
HbA% (×0.01)	-1.41 (-2.87, 0.43)	**0.023**	-		-	
HbS% (×0.01)	0.50 (-1.04, 2.40)	0.466	-		-	

CBF_i, cerebral blood flow index; Hb, hemoglobin; HbA%, percent hemoglobin A out of total hemoglobin; HbS%, percent hemoglobin S out of total hemoglobin; OEF, oxygen extraction fraction; CBV, cerebral blood volume; $CMRO_{2i}$, cerebral metabolic rate of oxygen. Hb variables (Hb, HbA %, HbS % were not modeled together due to multicollinearity; [1]Multivariable models considering Hb with other predictors; [2]Multivariable models considering HbA with other predictors.
